# Machine learning-driven identification and immunohistochemical validation of an integrated immune-inflammatory phenotype for disease-free survival stratification in breast cancer

**DOI:** 10.3389/fimmu.2026.1836156

**Published:** 2026-06-18

**Authors:** Shanshan Han, Lin Ran, Zhaoan Lian, Yong Tian, Li Qin, Yingchun Xiang, Xiaohao Yan, Chengyu Shui, Cheng Huang

**Affiliations:** 1 Affliated Hospital of Anhui West Health Vocational College, Lu’an, Anhui, China; 2 Department of Obstetrics and Gynecology, Central Hospital of Enshi Tujia and Miao Autonomous Prefecture, Enshi Clinical College of Wuhan University, Enshi, China; 3 Chengdu Huake Biology Research Center, Chengdu, China

**Keywords:** breast cancer, disease-free survival, integrated immune phenotype, random survival forest, systemic immune-inflammation index, tumor-infiltrating lymphocytes

## Abstract

**Background:**

Recurrence risk in breast cancer remains heterogeneous, and conventional clinicopathological variables may not fully capture the contribution of immune-related factors. We compared multiple survival modeling strategies and evaluated whether an integrated immune-inflammatory phenotype could improve disease-free survival (DFS) stratification.

**Methods:**

This retrospective single-center study included 503 patients with surgically treated breast cancer between January 2020 and December 2025. Stromal tumor-infiltrating lymphocytes (TILs) were assessed from pathological sections, and the systemic immune-inflammation index (SII) was calculated from pre-treatment blood counts. An integrated immune phenotype was defined as favorable (high TILs/low SII), poor (low TILs/high SII), or intermediate (all remaining combinations). A base clinical Cox model, an immune-extended Cox model, LASSO-Cox, CoxBoost, and random survival forest (RSF) were compared using C-index, time-dependent area under the curve (AUC), integrated Brier score (IBS), and decision curve analysis. Conventional survival analyses, restricted cubic spline analysis, and immunohistochemical validation with CD8 and CD163 staining were also performed.

**Results:**

During follow-up, 107 patients (21.3%) experienced a DFS event. RSF achieved the best overall performance, with time-dependent AUCs of 0.867, 0.880, 0.879, 0.893, and 0.911 at 12, 24, 36, 48, and 60 months, respectively, and the lowest IBS (0.100). In the RSF model, pathological N stage was the most important predictor, followed by SII, integrated immune phenotype, Ki-67, and lymphovascular invasion. Kaplan–Meier analysis showed no significant DFS difference according to TIL category alone, whereas high SII and the poor integrated immune phenotype were associated with significantly worse DFS. In the final multivariable Cox model, the poor phenotype remained independently associated with worse DFS compared with the favorable phenotype (hazard ratio 2.53, 95% confidence interval 1.39–4.60; *p* = 0.002). Immunohistochemical validation showed higher CD8+ cell density, lower CD163+ cell density, and a higher CD8/CD163 ratio in the favorable phenotype than in the poor phenotype.

**Conclusion:**

RSF provided the best prognostic performance in this cohort. SII and the integrated immune phenotype emerged as clinically relevant predictors, and the integrated phenotype showed tissue-level biological support. Combining machine learning-based survival modeling with pragmatic immune-inflammatory markers may improve recurrence risk stratification in breast cancer.

## 1 Introduction

Breast cancer remains the most commonly diagnosed malignancy among women worldwide and continues to impose a substantial global health burden. GLOBOCAN 2020 estimated 2.3 million new cases and approximately 685,000 related deaths, underscoring the scale of the disease (1, 2). Although substantial progress has been made in surgery, systemic therapy, and subtype-guided treatment, recurrence risk after primary treatment is still far from uniform. In daily practice, clinicians often encounter patients with apparently similar clinicopathological features but very different outcomes. This gap suggests that conventional prognostic factors do not fully capture the biological complexity underlying disease progression (1, 3).

This heterogeneity is shaped not only by tumor-intrinsic characteristics, but also by the interaction between the tumor and the host immune system (4–6). In breast cancer, stromal tumor-infiltrating lymphocytes (TILs) are among the most extensively studied local immune markers, and standardized recommendations have improved the consistency of their assessment (7). Higher TIL levels have repeatedly been associated with better therapeutic response and more favorable outcomes, especially in triple-negative and HER2-positive disease (8–11). At the same time, the prognostic value of TILs is not uniform across all clinical settings, and their significance may be less clear in heterogeneous real-world cohorts.

Systemic inflammatory markers offer another perspective. Among them, the systemic immune-inflammation index (SII), derived from platelets, neutrophils, and lymphocytes, has received increasing attention because it captures the balance between protumor inflammatory activation and impaired antitumor immune surveillance (5, 12, 13). In breast cancer, elevated SII has been associated with poorer survival and more aggressive disease behavior (12). Still, as a blood-based marker, SII offers only a broad reflection of host status and cannot by itself describe the local immune architecture of the tumor.

Taken together, these observations suggest that combining local immune infiltration with systemic inflammatory burden may yield more informative prognostic stratification than considering either dimension alone. Yet, in most previous studies, these markers have been examined separately, and simple integrative approaches that can be readily applied in routine clinical datasets remain limited (14–16). Another unresolved issue is methodological. Most prognostic studies in breast cancer still rely mainly on conventional Cox regression. Although clinically interpretable, such models may be less suited to capturing nonlinear associations or complex interactions, especially for inflammatory variables such as SII. Machine learning-based survival models, including random survival forest (RSF), may be better able to accommodate such complexity without imposing strong parametric assumptions (15, 17, 18). Even so, a model is more convincing when its predictive signal can be connected to biologically meaningful tissue-level findings.

Against this background, we compared several survival modeling strategies, including conventional Cox-based models, LASSO-Cox, CoxBoost, and RSF, to identify an appropriate framework for disease-free survival (DFS) prediction in a municipal hospital breast cancer cohort. We then examined the prognostic relevance of SII and an integrated immune phenotype combining stromal TILs and SII, and further evaluated whether this composite phenotype corresponded to a biologically distinct immune state by immunohistochemical assessment of CD8 and CD163. Through this approach, we sought to improve postoperative risk stratification while also linking predictive modeling to interpretable immune biology.

## 2 Materials and methods

### 2.1 Study design and study population

This single-center retrospective cohort study included consecutive patients with pathologically confirmed breast cancer who underwent surgical treatment at a municipal hospital between January 2020 and December 2025. Patients were included if they met all of the following criteria: (1) pathologically confirmed invasive breast cancer; (2) surgical treatment performed during the predefined study period; (3) complete baseline clinicopathological information available in the hospital information system and pathology database; (4) available pre-treatment peripheral blood test results for calculation of inflammatory indices; and (5) evaluable stromal TIL assessment from pathological sections. Patients were excluded if they met any of the following criteria: (1) metastatic disease at initial diagnosis; (2) concurrent hematologic disease or autoimmune disorder likely to substantially affect systemic inflammatory markers; (3) active infection at the time of blood sampling; or (4) missing follow-up data precluding disease-free survival (DFS) assessment. After application of these criteria, 503 patients were included in the final analysis.

This study was conducted in accordance with the Declaration of Helsinki. The study was approved by the Ethics Committee of Enshi Tujia & Miao Autonomous Prefecture Central Hospital (Approval No: 2025039). Given the retrospective nature of the study and the use of anonymized/de-identified clinical data, the requirement for informed consent was waived by the committee.

### 2.2 Data collection and variable definition

Clinical, pathological, laboratory, treatment, and follow-up data were extracted from the hospital information system and pathology database. Baseline variables included age, body mass index (BMI), menopausal status, histologic subtype, pathological T stage, pathological N stage, histologic grade, molecular subtype, Ki-67, lymphovascular invasion, PD-L1 status, type of breast surgery, receipt of neoadjuvant therapy, and receipt of adjuvant chemotherapy.

Pre-treatment peripheral blood parameters included neutrophil count, lymphocyte count, platelet count, albumin, and C-reactive protein. The systemic immune-inflammation index (SII) was calculated as platelet count × neutrophil count/lymphocyte count. Additional inflammatory indices, including neutrophil-to-lymphocyte ratio, platelet-to-lymphocyte ratio, systemic inflammation response index, and prognostic nutritional index, were also derived for supplementary analyses.

Pathological T stage was collapsed into three categories (T1, T2, and T3–4) for regression modeling because of the very small number of T4 cases. Pathological N stage was categorized as N0, N1, N2, and N3. Molecular subtype was classified as HR+/HER2−, HER2+, and triple-negative breast cancer (TNBC) according to routine immunohistochemical assessment of estrogen receptor, progesterone receptor, and HER2 status. Ki-67 was analyzed as both a continuous variable and a categorical variable, with high Ki-67 defined as ≥20%.

### 2.3 Evaluation of stromal tumor-infiltrating lymphocytes and integrated immune phenotype

Stromal TILs were assessed from routine pathological sections and recorded as the percentage of stromal area occupied by mononuclear inflammatory cells within the invasive tumor area. For categorical analyses, stromal TILs were dichotomized using a predefined cutoff of 20%, yielding high TILs (≥20%) and low TILs (<20%).

SII was calculated as platelet count × neutrophil count/lymphocyte count. In the primary categorical analysis, SII was dichotomized at the cohort median. We selected the median rather than an outcome-optimized cutoff because no universally accepted SII threshold has been established in breast cancer, and previously reported cutoffs vary substantially across studies depending on population, treatment setting, and statistical approach. Prior breast cancer studies have used ROC-derived or other data-driven thresholds, including values such as 514, 560, 601.7, and 755.1, whereas other studies have used cohort-specific median values such as 430 or 446 (19–21).

Given this heterogeneity, and to reduce overfitting and preserve balanced group sizes in this single-center retrospective cohort, we used the cohort median as a pragmatic and reproducible cutoff for the main categorical analysis. To avoid relying exclusively on dichotomization, SII was also analyzed as a continuous variable, and its association with disease-free survival was further examined using restricted cubic spline analysis. This strategy was chosen to retain clinical interpretability while also assessing potential nonlinearity (22).

To jointly characterize local immune infiltration and systemic inflammatory burden, an integrated immune phenotype was constructed by combining TIL category and SII category. This framework was designed to distinguish concordant favorable immune-inflammatory conditions, concordant unfavorable conditions, and discordant intermediate states. Specifically, the favorable phenotype was defined as high TILs and low SII, reflecting relatively preserved local antitumor immunity together with lower systemic inflammatory burden. The poor phenotype was defined as low TILs and high SII, reflecting limited local immune infiltration together with an adverse systemic inflammatory profile. The remaining two discordant combinations (high TILs/high SII and low TILs/low SII) were grouped as the intermediate phenotype. This categorization was intended as a pragmatic clinical stratification scheme rather than a comprehensive biological taxonomy of the tumor immune microenvironment.

### 2.4 Outcome definition

The primary endpoint was DFS. DFS time was calculated from the date of surgery to the date of documented recurrence, progression, death, or last follow-up, whichever occurred first. Patients without an event were censored at the last available follow-up.

### 2.5 Immunohistochemical validation

To determine whether the integrated immune phenotype corresponded to a biologically distinct tumor immune microenvironment, immunohistochemical validation was performed using formalin-fixed paraffin-embedded tumor specimens from representative cases in the favorable and poor phenotype groups (25 cases per group). Cases were selected based on the availability of adequate archived tissue and representative invasive tumor areas.

Serial 4-μm sections were deparaffinized, rehydrated, and subjected to heat-induced epitope retrieval using high-pH retrieval buffer at 97 °C for 20 min, followed by endogenous peroxidase blocking. Sections were incubated with primary antibodies against CD8 (clone C8/144B, Agilent/Dako, IR623, ready-to-use) and CD163 (clone 10D6, Leica/Novocastra, NCL-CD163, 1:200). PD-L1 staining was performed using the PD-L1 IHC 22C3 pharmDx assay on the Autostainer Link 48 platform according to the manufacturer’s instructions. Signal detection was carried out using the EnVision FLEX system with DAB as chromogen, and slides were counterstained with hematoxylin.

CD8 was used to reflect cytotoxic T-cell infiltration, whereas CD163 was used as a marker of macrophage-dominant immune suppression. Stained slides were independently reviewed by two pathologists blinded to clinical outcome and integrated phenotype classification. Representative invasive tumor areas were identified at low magnification, and five non-overlapping high-power fields (×400) were selected while avoiding necrosis, artifact, and extensive *in situ* components. Positively stained cells were manually counted, and mean cell density was expressed as cells/mm². The CD8/CD163 ratio was calculated as an index of the balance between cytotoxic lymphocytic infiltration and macrophage-associated immune suppression. PD-L1 positivity was defined as a combined positive score (CPS) ≥10. Quantitative variables were compared using the Wilcoxon rank-sum test, and categorical variables were compared using Pearson’s chi-square test or Fisher’s exact test, as appropriate.

### 2.6 Statistical analysis

#### 2.6.1 Conventional survival analyses

Continuous variables are presented as mean ± standard deviation when approximately normally distributed and as median (interquartile range) when non-normally distributed; categorical variables are presented as frequencies with percentages. Group comparisons between patients with and without DFS events were performed using the Wilcoxon rank-sum test for continuous variables and Pearson’s chi-square test or Fisher’s exact test for categorical variables, as appropriate. Comparisons across integrated immune phenotype groups were performed using the Kruskal-Wallis test for continuous variables and Pearson’s chi-square test for categorical variables.

To provide clinically interpretable analyses complementary to the machine learning results, DFS was also evaluated using the Kaplan–Meier method and compared using the log-rank test according to TIL category, SII category, and integrated immune phenotype. To assess the functional form of the association between SII and DFS, restricted cubic spline analysis was performed within a Cox proportional hazards regression framework using 4 knots. The spline model was adjusted for age, BMI, menopausal status, pathological T stage, pathological N stage, histologic grade, molecular subtype, Ki-67, lymphovascular invasion, breast surgery, adjuvant chemotherapy, and TIL category. A final multivariable Cox proportional hazards model was then constructed to provide an interpretable clinical complement to the machine learning analyses. This model included core clinicopathological variables together with the integrated immune phenotype. Hazard ratios (HRs) and 95% confidence intervals (CIs) were reported. The proportional hazards assumption was assessed using Schoenfeld residuals.

#### 2.6.2 Subgroup analyses

Exploratory subgroup analyses were performed to further assess the prognostic value of the integrated immune phenotype across pathological N stage, molecular subtype, breast surgery, adjuvant chemotherapy, and neoadjuvant therapy. Within each subgroup, hazard ratios and 95% confidence intervals for the poor phenotype compared with the favorable phenotype were estimated using Cox proportional hazards models adjusted according to the subgroup-analysis framework, excluding the corresponding stratification variable itself within each subgroup. *p* values for interaction were derived from likelihood ratio tests comparing models with and without the interaction term. These analyses were considered exploratory.

#### 2.6.3 Machine learning-based survival modeling

To identify the most suitable prognostic modeling strategy while reducing optimistic bias in this moderate-sized single-center cohort, we compared multiple survival prediction approaches using repeated stratified 10-fold cross-validation. The evaluated models included a base clinical Cox model, an immune-extended Cox model, LASSO-Cox, CoxBoost, and random survival forest (RSF). The base clinical model served as an interpretable benchmark and included age, BMI, menopausal status, pathological T stage, pathological N stage, histologic grade, molecular subtype, Ki-67, lymphovascular invasion, breast surgery, and adjuvant chemotherapy. The immune-extended model additionally incorporated immune-related variables. LASSO-Cox and CoxBoost were included as penalized Cox-based approaches to improve model stability in the presence of correlated predictors, whereas RSF was included as a nonparametric ensemble survival method capable of capturing nonlinear associations and higher-order interactions without requiring prespecification of their functional forms.

Candidate predictors were prespecified on clinical and biological grounds rather than generated through broad data-driven feature expansion. Because some clinicopathological strata were sparse, very infrequent categories were collapsed where appropriate within the machine-learning pipeline to preserve numerical stability. In each cross-validation iteration, model fitting was performed in the training folds and performance was evaluated using out-of-fold predictions. LASSO-Cox used 10-fold cross-validation within the training folds to determine the penalty parameter, and CoxBoost used penalty optimization followed by 10-fold cross-validation to determine the optimal number of boosting steps. Predictive performance was summarized using Harrell’s C-index, time-dependent receiver operating characteristic analysis, and the integrated Brier score (IBS). Time-dependent area under the ROC curve (AUC) values were estimated at 12, 24, 36, 48, and 60 months. Clinical utility was assessed by decision curve analysis (DCA) using out-of-fold predicted 36-month recurrence risk. Model interpretation was subsequently performed using SHAP analysis for the final RSF model.

Given the moderate sample size and limited event number, repeated stratified 10-fold cross-validation was considered more appropriate than a single random train-validation split, because it preserves statistical efficiency and provides a more stable internal assessment of generalization performance.

All statistical analyses were performed using R software. All tests were two-sided, and a *p* value <0.05 was considered statistically significant.

## 3 Results

### 3.1 Patient characteristics

**Figure 1:**
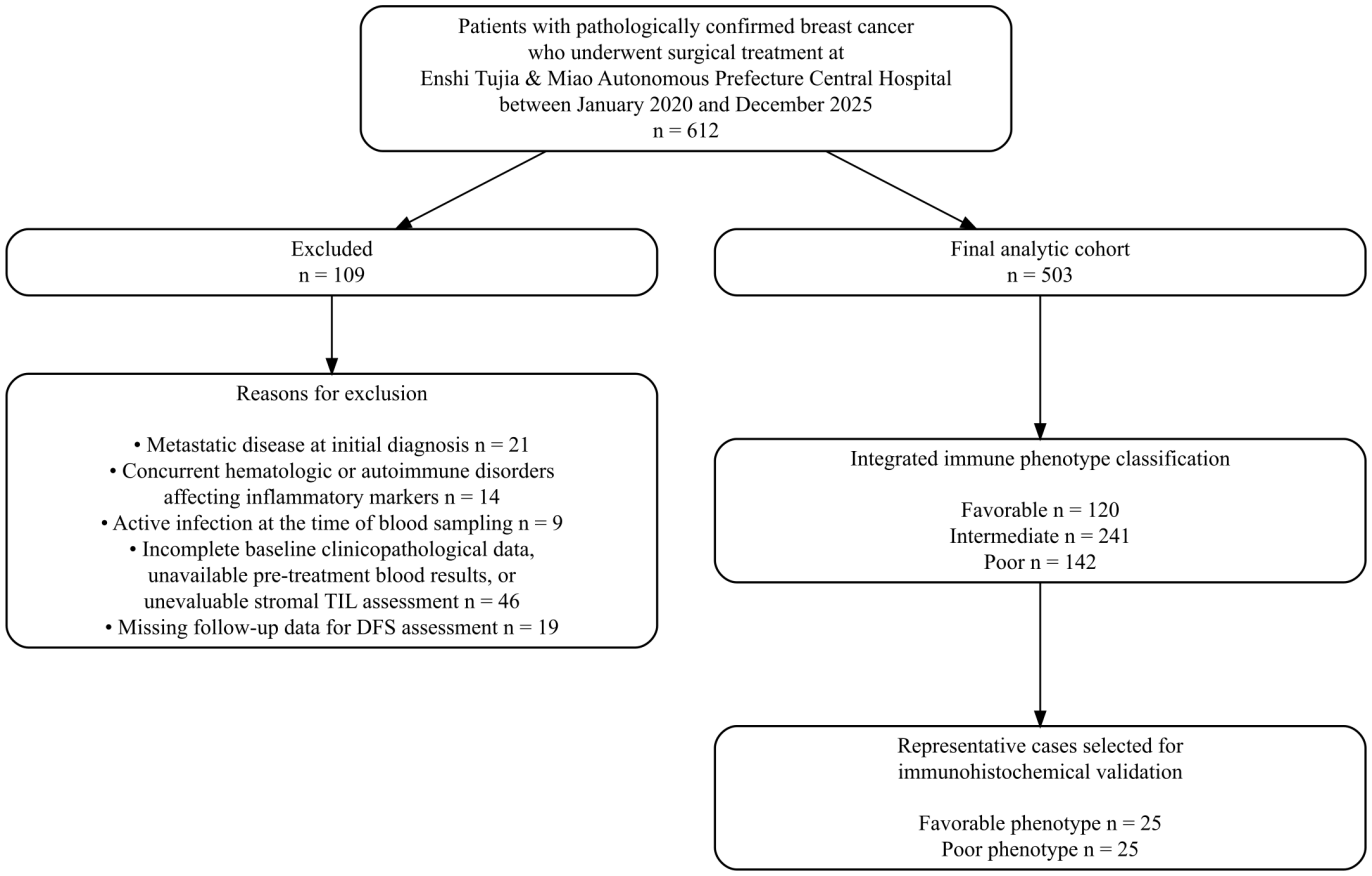
Flow chart of patient selection. Consecutive patients with pathologically confirmed breast cancer who underwent surgical treatment between January 2020 and December 2025 were screened. Patients were excluded because of metastatic disease at initial diagnosis, hematologic or autoimmune disorders likely to affect inflammatory markers, active infection at the time of blood sampling, incomplete baseline clinicopathological or laboratory data, unevaluable stromal TIL assessment, or missing follow-up data for disease-free survival assessment. A total of 503 patients were included in the final analytic cohort. Patients were further classified into favorable, intermediate, and poor integrated immune phenotype groups, and representative favorable and poor phenotype cases were selected for immunohistochemical validation.

A total of 503 patients with breast cancer were included in the final analysis (**Figure 1**), among whom 107 (21.3%) experienced a disease-free survival (DFS) event during follow-up. Baseline characteristics according to DFS event status are summarized in **Table 1**, with additional variables shown in **Supplementary Table 1**.

**Table 1:** Baseline clinicopathological and immune-inflammatory characteristics according to disease-free survival event status.

Characteristic	Overall N = 503	No event N = 396	Event N = 107	*p*-value
Age, years	53.49 ± 10.89	53.29 ± 10.94	54.20 ± 10.71	0.5
Body mass index, kg/m²	24.83 ± 3.62	24.81 ± 3.59	24.90 ± 3.76	0.7
Tumor size, cm	2.50 (1.90, 3.24)	2.46 (1.84, 3.16)	2.69 (2.16, 3.38)	0.055
Stromal TILs, %	18.00 (11.00, 27.00)	18.50 (11.00, 28.00)	17.00 (8.00, 27.00)	0.3
Systemic immune-inflammation index	477.80 (370.42, 633.97)	466.01 (354.59, 618.59)	561.92 (421.36, 684.61)	<0.001
Menopausal status				0.6
Premenopausal	210 (42%)	168 (42%)	42 (39%)	
Postmenopausal	293 (58%)	228 (58%)	65 (61%)	
Pathological T stage				0.076
T1	144 (29%)	122 (31%)	22 (21%)	
T2	357 (71%)	273 (69%)	84 (79%)	
T3-4	2 (0.4%)	1 (0.3%)	1 (0.9%)	
Pathological N stage				<0.001
N0	335 (67%)	286 (72%)	49 (46%)	
N1	83 (17%)	61 (15%)	22 (21%)	
N2	57 (11%)	31 (7.8%)	26 (24%)	
N3	28 (5.6%)	18 (4.5%)	10 (9.3%)	
Histologic grade				0.072
1	47 (9.3%)	42 (11%)	5 (4.7%)	
2	245 (49%)	196 (49%)	49 (46%)	
3	211 (42%)	158 (40%)	53 (50%)	
Molecular subtype				0.5
HR+/HER2-	284 (56%)	229 (58%)	55 (51%)	
HER2+	114 (23%)	87 (22%)	27 (25%)	
TNBC	105 (21%)	80 (20%)	25 (23%)	
Ki-67 category				0.3
Low	153 (30%)	125 (32%)	28 (26%)	
High	350 (70%)	271 (68%)	79 (74%)	
Lymphovascular invasion	86 (17%)	52 (13%)	34 (32%)	<0.001
TIL category				0.2
High (>=20%)	230 (46%)	187 (47%)	43 (40%)	
Low (<20%)	273 (54%)	209 (53%)	64 (60%)	
PD-L1 status				0.8
Negative	404 (80%)	317 (80%)	87 (81%)	
Positive	99 (20%)	79 (20%)	20 (19%)	
SII category				<0.001
Low	251 (50%)	214 (54%)	37 (35%)	
High	252 (50%)	182 (46%)	70 (65%)	
Integrated immune phenotype				0.001
Favorable	120 (24%)	102 (26%)	18 (17%)	
Intermediate	241 (48%)	197 (50%)	44 (41%)	
Poor	142 (28%)	97 (24%)	45 (42%)	
Received neoadjuvant therapy	147 (29%)	119 (30%)	28 (26%)	0.4
Breast surgery				0.3
Breast-conserving	122 (24%)	100 (25%)	22 (21%)	
Mastectomy	381 (76%)	296 (75%)	85 (79%)	
Adjuvant chemotherapy	462 (92%)	361 (91%)	101 (94%)	0.3

Continuous variables are presented as mean ± standard deviation or median (interquartile range), as appropriate, and categorical variables are presented as n (%). *p* values were calculated using Student’s t-test or the Wilcoxon rank-sum test for continuous variables, and Pearson’s chi-square test or Fisher’s exact test for categorical variables, as appropriate.

DFS, disease-free survival; TILs, tumor-infiltrating lymphocytes; SII, systemic immune-inflammation index; PD-L1, programmed death-ligand 1.

Compared with patients without a DFS event, those with events had significantly higher baseline systemic immune-inflammation index (SII) levels and a greater proportion of high SII status (both *p* < 0.001). The distribution of the integrated immune phenotype also differed significantly between groups (*p* = 0.001), with the poor phenotype being more frequent in patients who experienced DFS events. Among conventional clinicopathological variables, nodal stage and lymphovascular invasion were significantly associated with event status (both *p* < 0.001), whereas tumor size, pathological T stage, and histologic grade showed only borderline differences. No significant differences were observed for age, body mass index, menopausal status, stromal TIL category, PD-L1 status, molecular subtype, Ki-67 category, neoadjuvant therapy, surgery type, or adjuvant chemotherapy.

In supplementary analyses, neutrophil-to-lymphocyte ratio, platelet-to-lymphocyte ratio, and systemic inflammation response index were also significantly higher in patients with DFS events, whereas receptor status, histologic subtype, albumin, C-reactive protein, and prognostic nutritional index were not significantly associated with event status (**Supplementary Table 1**).

### 3.2 Characteristics according to integrated immune phenotype

Baseline characteristics stratified by integrated immune phenotype are shown in **Supplementary Table 2**. Of the 503 patients, 120 (24%) were classified as favorable, 241 (48%) as intermediate, and 142 (28%) as poor.

As expected, stromal TIL levels and SII differed significantly across phenotype groups (both *p* < 0.001). In addition, histologic grade, molecular subtype, lymphovascular invasion, and PD-L1 status also varied significantly across groups. In contrast, age, body mass index, tumor size, pathological T stage, pathological N stage, Ki-67 category, neoadjuvant therapy, breast surgery, and adjuvant chemotherapy did not differ significantly among phenotype groups.

### 3.3 Cross-validated model performance comparison

**Figure 2:**
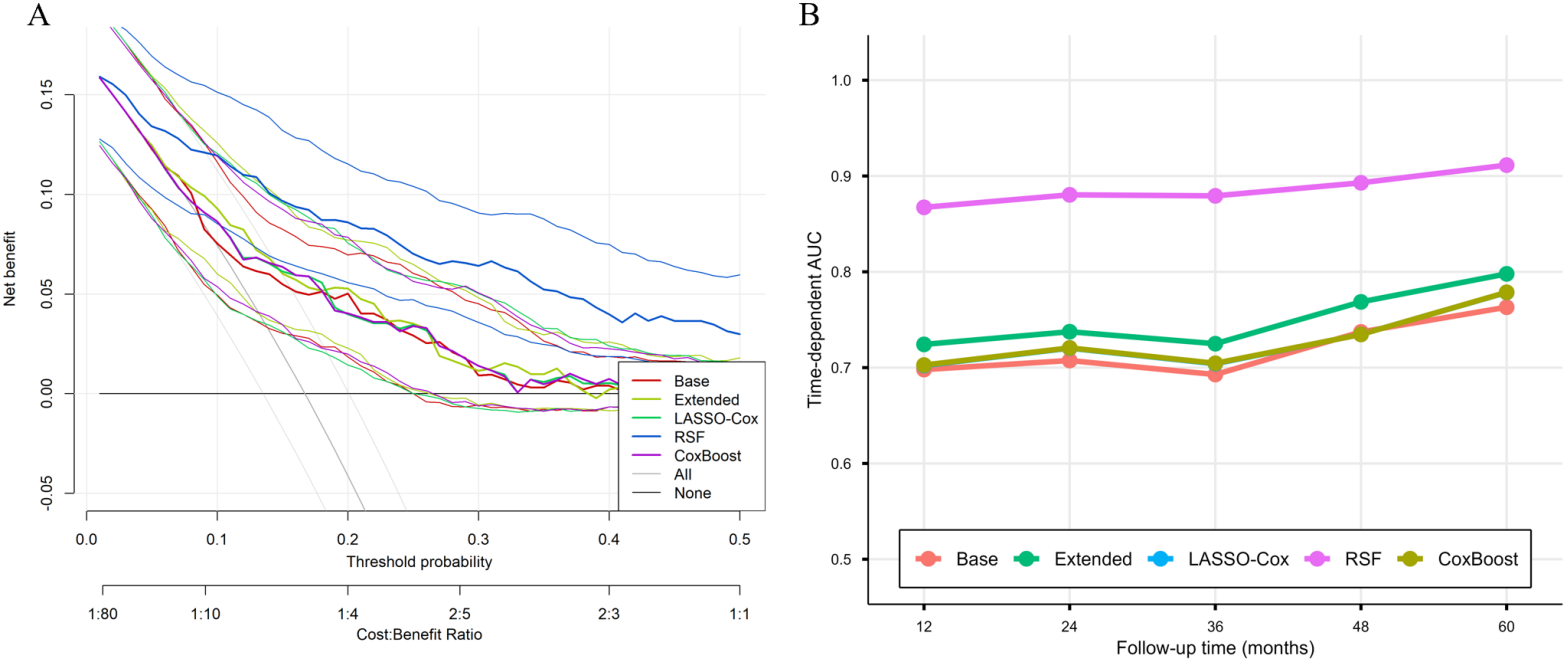
Machine learning-based model performance comparison for disease-free survival prediction. **(A)** Decision curve analysis comparing the clinical utility of the five models across threshold probabilities. **(B)** Dynamic AUC curves comparing the base model, extended model, LASSO-Cox, CoxBoost, and random survival forest (RSF).

To compare predictive performance across modeling strategies, we evaluated a conventional clinical model (Base), an immune-extended model (Extended), and three machine learning-based survival models, including LASSO-Cox, CoxBoost, and random survival forest (RSF) using repeated stratified 10-fold cross-validation. Model performance is summarized in **Table 2** and **Figure 2**.

**Table 2:** Machine learning model performance comparison for disease-free survival prediction.

Model	C-index	12-month AUC	24-month AUC	36-month AUC	48-month AUC	60-month AUC	IBS
Base	0.697	0.698	0.707	0.693	0.737	0.763	0.136
Extended	0.726	0.724	0.737	0.725	0.769	0.798	0.133
LASSO-Cox	0.705	0.702	0.720	0.704	0.735	0.778	0.136
RSF	0.853	0.867	0.880	0.879	0.893	0.911	0.100
CoxBoost	0.704	0.703	0.721	0.705	0.735	0.779	0.136

Predictive performance was compared across five models, including the base clinical model, the immune-extended model, LASSO-Cox, random survival forest (RSF), and CoxBoost. Discrimination was assessed using Harrell’s C-index and time-dependent area under the receiver operating characteristic curve (AUC) at 12, 24, 36, 48, and 60 months. Overall prediction error was evaluated using the integrated Brier score (IBS), with lower values indicating better performance.

AUC, area under the receiver operating characteristic curve; IBS, integrated Brier score; RSF, random survival forest.

Across all evaluated follow-up time points, RSF consistently achieved the best discriminative performance. Its time-dependent AUCs were 0.867, 0.880, 0.879, 0.893, and 0.911 at 12, 24, 36, 48, and 60 months, respectively, clearly outperforming the other models (**Table 2**, **Figure 2B**). In addition, RSF showed the lowest integrated Brier score (IBS = 0.100), together with the highest cross-validated C-index(C-index = 0.853), indicating the best overall prediction accuracy among the tested approaches. The Extended model consistently outperformed the Base model across all follow-up times, suggesting that incorporation of immune-related information improved predictive performance even within a conventional regression-based framework. By contrast, LASSO-Cox and CoxBoost provided only modest improvements over the Base model and remained inferior to RSF.

Decision curve analysis demonstrated a similar pattern (**Figure 2A**). Over a broad range of clinically relevant threshold probabilities, RSF yielded the highest net benefit, whereas the Extended model generally performed better than the Base model and the other machine learning approaches. Taken together, these findings support RSF as the optimal prognostic modeling strategy in this cohort.

### 3.4 Model interpretation using SHAP analysis

**Figure 3:**
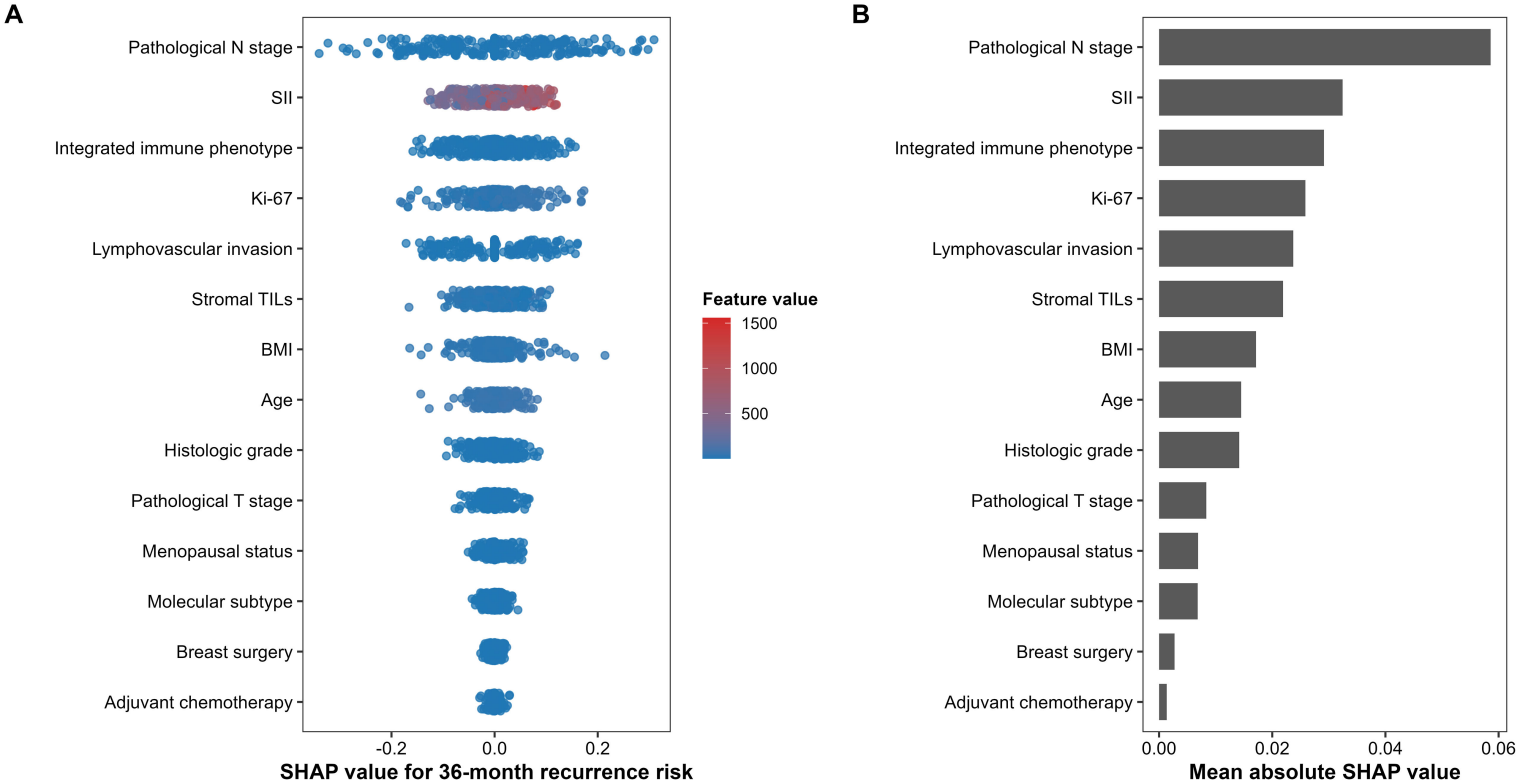
SHAP-based interpretation of the random survival forest model (RSF) for 36-month recurrence risk prediction. **(A)** SHAP summary plot showing the distribution and direction of individual variable contributions to the RSF-predicted 36-month recurrence risk. **(B)** Mean absolute SHAP value plot showing the overall importance ranking of predictors in the RSF model.

Given the superior predictive performance of the RSF model, we next used SHAP analysis to improve model interpretability and to identify the variables contributing most strongly to the predicted 36-month recurrence risk (**Figure 3**).

The SHAP summary plot showed that pathological N stage had the largest overall contribution to RSF-based recurrence prediction, followed by SII, the integrated immune phenotype, Ki-67, and lymphovascular invasion (**Figure 3A**). Stromal TILs also contributed to the model, although their impact was less pronounced than that of SII and the integrated immune phenotype. In contrast, menopausal status, molecular subtype, breast surgery, and adjuvant chemotherapy showed relatively limited contributions to the RSF-predicted 36-month recurrence risk.

The mean absolute SHAP value plot showed a consistent ranking (**Figure 3B**). Pathological N stage was the dominant predictor, while SII and the integrated immune phenotype ranked among the most influential immune-related variables, ahead of stromal TILs alone. These findings suggest that recurrence risk in this cohort was driven not only by tumor burden and invasive pathological characteristics, but also by systemic inflammatory status and combined immune stratification.

Taken together, the SHAP analysis supported the biological and clinical relevance of the integrated immune-inflammatory framework and provided an interpretable explanation for the superior predictive performance of the RSF model.

### 3.5 Restricted cubic spline analysis of SII and disease-free survival

**Figure 4:**
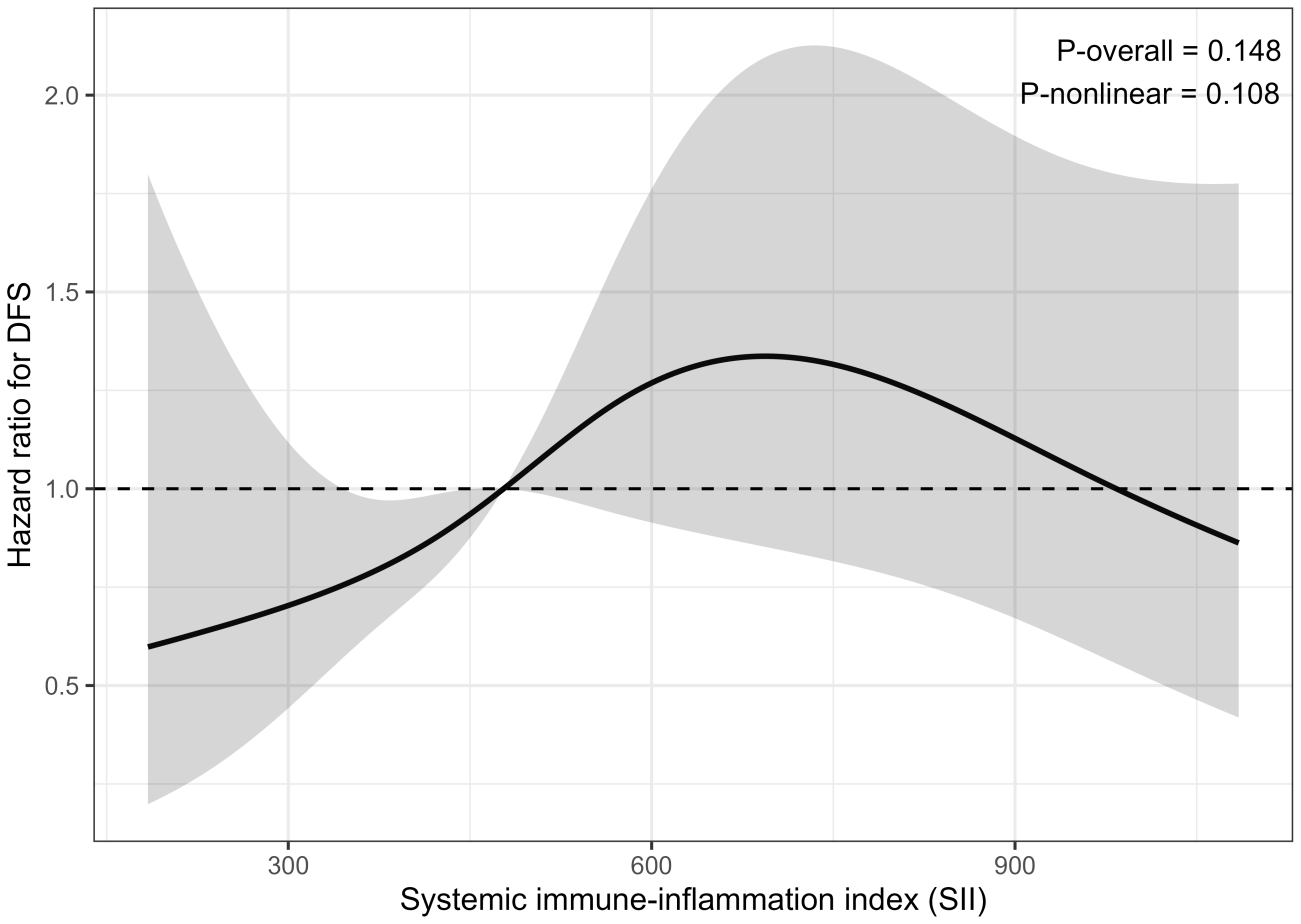
Restricted cubic spline analysis of the association between systemic immune-inflammation index and disease-free survival risk. The solid line represents the adjusted hazard ratio, and the shaded area indicates the 95% confidence interval. The model was adjusted for age, BMI, menopausal status, pathological T stage, pathological N stage, histologic grade, molecular subtype, Ki-67, lymphovascular invasion, breast surgery, adjuvant chemotherapy, and TIL category. The overall association between SII and DFS was not statistically significant (*p*-overall = 0.148), and the nonlinear component was also not statistically significant (*p*-nonlinear = 0.108).

Because SII ranked among the more important predictors in the RSF model, we further explored its continuous relationship with DFS using restricted cubic spline analysis (**Figure 4**). The spline curve suggested a non-monotonic trend between SII and DFS risk, with the estimated hazard ratio increasing across the intermediate-to-high SII range and then attenuating at the upper tail. However, neither the overall association nor the nonlinear component reached statistical significance (*p*-overall = 0.148; *p*-nonlinear = 0.108). Confidence intervals widened at the extremes of the SII distribution, indicating greater uncertainty in those regions. These findings suggest a possible complex pattern in the association between systemic inflammatory burden and DFS, although the spline analysis did not provide statistically significant evidence for a nonlinear relationship in this cohort.

### 3.6 Kaplan–Meier survival analyses of immune-inflammatory stratification

**Figure 5:**
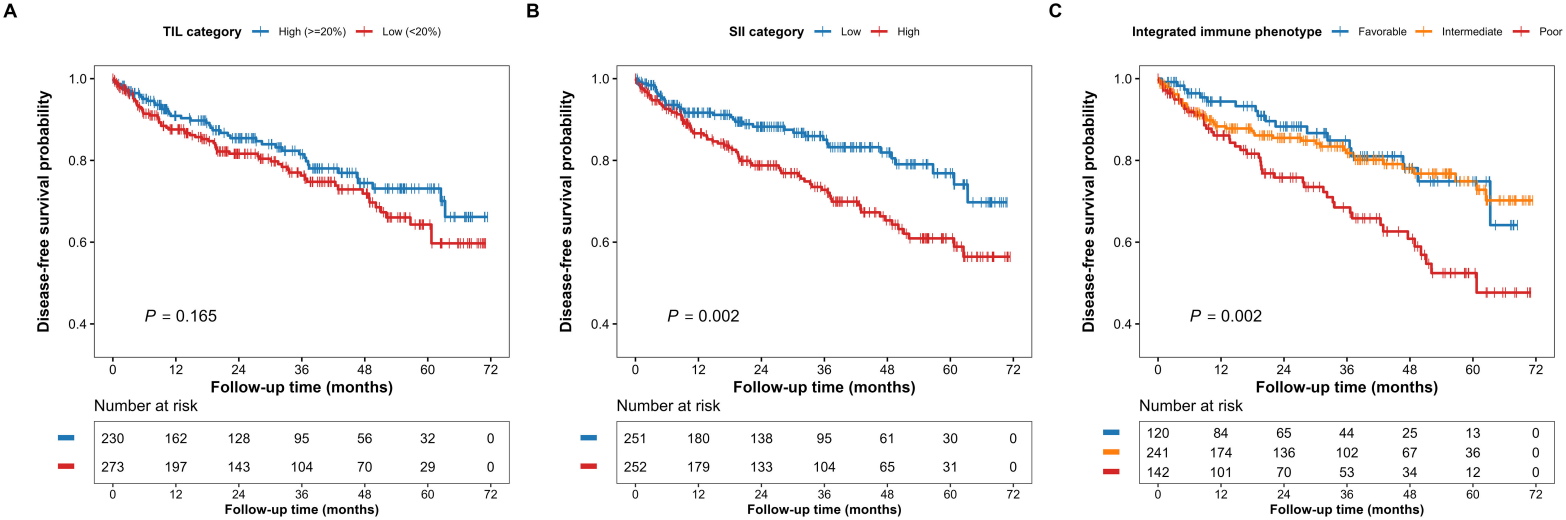
Kaplan–Meier curves for disease-free survival according to immune-inflammatory stratification. **(A)** TIL category. **(B)** SII category. **(C)** Integrated immune phenotype.

To provide clinically interpretable survival stratification, we performed Kaplan–Meier analyses according to TIL category, SII category, and the integrated immune phenotype (**Figure 5**).

DFS did not differ significantly according to TIL category alone (*p* = 0.165; **Figure 5A**). In contrast, patients with high SII had significantly worse DFS than those with low SII (*p* = 0.002; **Figure 5B**). When TILs and SII were integrated into a composite immune phenotype, survival separation became more pronounced (**Figure 5C**). Patients with the poor phenotype had the least favorable DFS, whereas those with the favorable phenotype had the best outcome (*p* = 0.002). These findings suggest that combined assessment of local immune infiltration and systemic inflammatory status provides stronger prognostic stratification than either component alone.

### 3.7 Final interpretable Cox proportional hazards model

To complement the machine learning results with a clinically interpretable model, we constructed a final multivariable Cox proportional hazards model incorporating core clinicopathological variables and the integrated immune phenotype (**Table 3**).

**Table 3:** Final interpretable Cox proportional hazards model for disease-free survival.

Variable	HR (95% CI)	*p* value
Pathological T stage: T2 vs T1	1.93 (1.11-3.35)	0.020
Pathological T stage: T3–4 vs T1	4.12 (0.50-34.18)	0.189
Pathological N stage: N1 vs N0	2.02 (1.16-3.51)	0.013
Pathological N stage: N2 vs N0	3.76 (2.22-6.38)	<0.001
Pathological N stage: N3 vs N0	3.13 (1.52-6.43)	0.002
Histologic grade: 2 vs 1	1.84 (0.72-4.70)	0.205
Histologic grade: 3 vs 1	3.25 (1.24-8.53)	0.017
Lymphovascular invasion: Yes vs No	1.93 (1.25-2.96)	0.003
Integrated immune phenotype: Intermediate vs Favorable	1.25 (0.71-2.19)	0.446
Integrated immune phenotype: Poor vs Favorable	2.53 (1.39-4.60)	0.002

Hazard ratios (HRs) and 95% confidence intervals (CIs) were estimated using a multivariable Cox proportional hazards model constructed to provide an interpretable clinical complement to the machine learning analyses. Reference categories were T1 for pathological T stage, N0 for pathological N stage, grade 1 for histologic grade, no lymphovascular invasion, and favorable phenotype for integrated immune phenotype.

HR, hazard ratio; CI, confidence interval; DFS, disease-free survival; TILs, tumor-infiltrating lymphocytes; SII, systemic immune-inflammation index.

Higher nodal burden remained a robust adverse prognostic factor. Compared with N0 disease, the hazard ratios (HRs) for DFS events were 2.02 (95% CI 1.16–3.51) for N1 disease, 3.76 (95% CI 2.22–6.38) for N2 disease, and 3.13 (95% CI 1.52–6.43) for N3 disease. Pathological T2 stage was also independently associated with worse DFS compared with T1 disease (HR 1.93, 95% CI 1.11–3.35, *p* = 0.020), whereas T3–4 disease did not reach statistical significance, likely reflecting the very small number of cases in this subgroup. Histologic grade 3, but not grade 2, remained significantly associated with poorer DFS (HR 3.25, 95% CI 1.24–8.53, *p* = 0.017). Lymphovascular invasion was also an independent adverse factor (HR 1.93, 95% CI 1.25–2.96, *p* = 0.003).

Importantly, compared with the favorable phenotype, the poor integrated immune phenotype remained independently associated with significantly worse DFS (HR 2.53, 95% CI 1.39–4.60, *p* = 0.002), whereas the intermediate phenotype was not significantly associated with outcome. These results indicate that the poor integrated immune phenotype provides clinically interpretable prognostic information beyond established clinicopathological variables. Exploratory subgroup analyses further showed no significant heterogeneity in the prognostic effect of the poor integrated immune phenotype across pathological N stage, molecular subtype, breast surgery, adjuvant chemotherapy, or neoadjuvant therapy (all *p* for interaction > 0.05; **Supplementary Table 3**).

### 3.8 Immunohistochemical validation of the integrated immune phenotype

**Figure 6:**
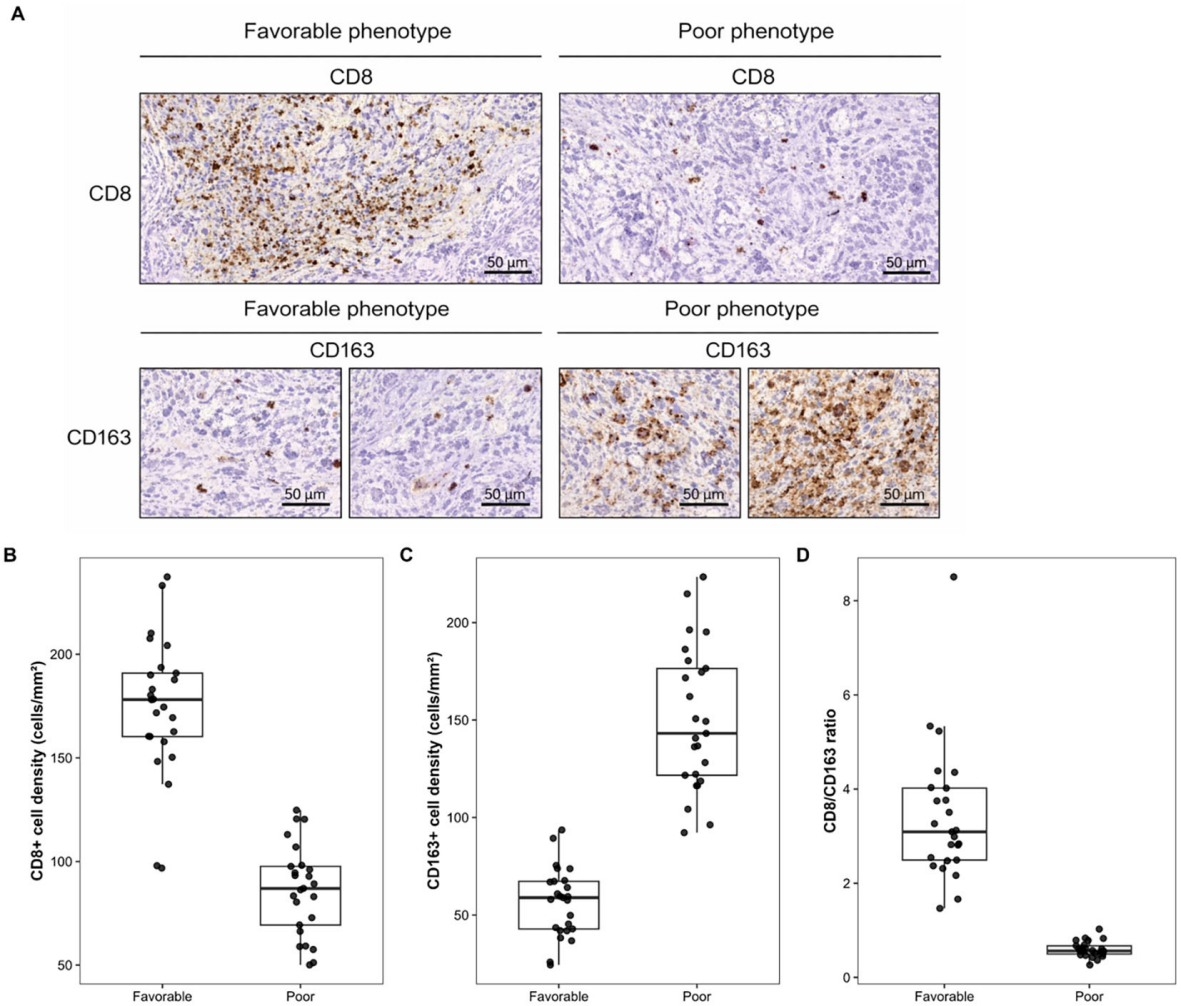
Immunohistochemical validation of the tumor immune microenvironment according to integrated immune phenotype. **(A)** Representative IHC images showing CD8 and CD163 staining patterns in favorable and poor phenotypes. **(B)** Comparison of CD8+ cell density. **(C)** Comparison of CD163+ cell density. **(D)** Comparison of the CD8/CD163 ratio.

To provide tissue-level support for the integrated immune phenotype, we performed immunohistochemical validation in representative tumor samples (**Figure 6**, **Table 4**).

**Table 4:** Immunohistochemical validation of tumor immune microenvironment features according to integrated immune phenotype.

Marker	Favorable phenotype (n = 25)	Poor phenotype (n = 25)	P value
CD8+ cell density (cells/mm²)	186 (142-233)	79 (51-118)	<0.001
CD163+ cell density (cells/mm²)	64 (42-95)	151 (108-196)	<0.001
CD8/CD163 ratio	2.76 (1.84-4.25)	0.56 (0.33-0.91)	<0.001
PD-L1 positive, n (%)	9 (36.0)	3 (12.0)	0.047

Data are presented as median (interquartile range) unless otherwise indicated. Comparisons between the favorable and poor phenotype groups were performed using the Wilcoxon rank-sum test for continuous variables and Pearson’s chi-square test or Fisher’s exact test for categorical variables, as appropriate. The favorable phenotype was defined as high TILs and low SII, whereas the poor phenotype was defined as low TILs and high SII.

TILs, tumor-infiltrating lymphocytes; SII, systemic immune-inflammation index; PD-L1, programmed death-ligand 1.

Representative images showed increased intratumoral CD8+ T-cell infiltration and reduced CD163+ macrophage infiltration in tumors with the favorable phenotype, whereas tumors with the poor phenotype showed the opposite pattern (**Figure 6A**). Quantitative analysis confirmed these differences. Compared with the poor phenotype group, the favorable phenotype group exhibited significantly higher CD8+ cell density (median 186 vs. 79 cells/mm^2^, *p* < 0.001), significantly lower CD163+ cell density (median 64 vs. 151 cells/mm^2^, *p* < 0.001), and a markedly higher CD8/CD163 ratio (median 2.76 vs. 0.56, *p* < 0.001) (**Figures 6B–D**, **Table 4**). PD-L1 positivity was also more frequent in the favorable phenotype group than in the poor phenotype group (36.0% vs. 12.0%, *p* = 0.047).

These findings support the biological relevance of the integrated immune phenotype, indicating that the poor phenotype corresponds to a more immune-suppressive tumor microenvironment characterized by lower cytotoxic T-cell infiltration and higher macrophage infiltration.

## 4 Discussion

In this retrospective cohort of 503 surgically treated patients with breast cancer, we found that an RSF-based survival model provided the best overall prognostic performance for DFS, outperforming conventional Cox models as well as LASSO-Cox and CoxBoost. More importantly, an integrated immune phenotype combining stromal TILs and SII provided clearer prognostic stratification than either marker alone, and this composite framework was supported by tissue-level differences in CD8, CD163, and the CD8/CD163 ratio. Taken together, these findings suggest that recurrence risk in breast cancer is shaped not only by tumor burden and conventional pathological aggressiveness, but also by the interaction between local antitumor immunity and host systemic inflammatory status.

A central finding of the present study is the superior performance of RSF. This result is biologically plausible and methodologically coherent. Breast cancer recurrence is influenced by heterogeneous and partly nonlinear interactions among nodal burden, proliferative activity, lymphovascular invasion, immune infiltration, and host inflammatory response (9, 23, 24). Classical Cox regression remains clinically interpretable, but it assumes proportional hazards and usually models continuous variables in a relatively constrained form unless nonlinear terms are prespecified (23). By contrast, RSF is a nonparametric ensemble method that can naturally accommodate nonlinear effects, high-order interactions, and complex covariate structures under censoring (25). Our SHAP analysis further showed that pathological N stage remained the dominant contributor to recurrence prediction, whereas SII and the integrated immune phenotype ranked ahead of stromal TILs alone, indicating that immune-inflammatory status contributed meaningful prognostic information beyond standard clinicopathological factors (23, 26, 27).

From a biological and clinical perspective, the most important finding is that the combination of TILs and SII was more informative than either component considered separately. Large pooled analyses and validation studies have shown that higher stromal TILs are most consistently associated with better response and improved survival in triple-negative and HER2-positive disease, whereas the association is weaker, absent, or even directionally different in luminal-HER2-negative tumors (10, 28, 29). Therefore, in a mixed real-world cohort such as ours, the overall prognostic signal of TILs alone may be diluted by subtype heterogeneity, treatment heterogeneity, and the fact that conventional H&E-based stromal TIL assessment does not distinguish functional immune subsets or spatial organization (11, 16, 29, 30). Our negative Kaplan–Meier result for TILs alone should thus not be interpreted as contradicting the TIL literature; rather, it likely indicates that a single tissue-level quantity is insufficient to summarize the prognostic meaning of the immune microenvironment across biologically diverse breast cancers. By contrast, SII showed a more robust relationship with outcome in our cohort, both in conventional survival analysis and in machine-learning ranking. This is also in line with the broader literature (14, 22). SII integrates neutrophils, platelets, and lymphocytes, thereby capturing a balance between protumor inflammatory activation and host immune competence (31). In breast cancer specifically, meta-analyses have reported that high pretreatment SII is associated with poorer OS and DFS, supporting its role as a low-cost systemic biomarker of adverse prognosis (14, 22).

The most clinically meaningful finding may be that combining TILs and SII generated stronger prognostic separation than either marker alone. Conceptually, this makes sense. A tumor with limited lymphocytic infiltration may not carry the same biological implication in a host with relatively quiescent systemic inflammation as in a host with marked inflammatory activation. Likewise, the favorable implication of a lymphocyte-rich tumor may be blunted when accompanied by a strongly adverse systemic immune-inflammatory profile. In other words, local immune contexture and systemic inflammatory tone are related but nonredundant dimensions (32, 33). Our integrated phenotype appears to capture this interaction in a pragmatic way: patients with high TILs/low SII had the best DFS, those with low TILs/high SII had the worst DFS, and the remaining combinations occupied an intermediate zone. Importantly, the poor phenotype remained independently associated with worse DFS in the final Cox model even after adjustment for nodal status, tumor size, grade, and lymphovascular invasion, indicating that this composite measure conveys information beyond conventional pathology (14).

The tissue-level validation strengthens the biological credibility of this composite framework. Compared with the poor phenotype, the favorable phenotype showed higher CD8+ cell density, lower CD163+ macrophage density, and a markedly higher CD8/CD163 ratio. This pattern is highly consistent with current understanding of the breast cancer immune microenvironment (34). CD8+ cytotoxic T cells are generally linked to effective antitumor immunity, while CD163 marks macrophages with an M2-like, immunosuppressive phenotype that is associated with tumor progression, matrix remodeling, and metastasis (34, 35). Several studies and meta-analyses have shown that high CD163+ tumor-associated macrophage infiltration is associated with poorer survival in breast cancer, whereas higher cytotoxic T-cell predominance or more favorable immune-cell ratios tend to accompany better outcomes (34, 36, 37). Our IHC results therefore do more than “illustrate” the phenotype categories; they suggest that the favorable and poor phenotypes correspond to meaningfully different immune ecologies at the tissue level.

The difference in PD-L1 positivity between phenotype groups is also noteworthy, although it should be interpreted cautiously. In breast cancer, PD-L1 expression often coexists with higher immune-cell infiltration and is especially enriched in more immunogenic subtypes such as TNBC (38). It may therefore reflect an adaptive immune resistance state rather than a uniformly adverse feature (39). Our finding that PD-L1 positivity was more frequent in the favorable phenotype is compatible with prior observations that PD-L1 can be positively associated with TIL abundance (40). In this context, higher PD-L1 in the favorable phenotype may indicate an inflamed microenvironment with active immune engagement rather than simple immune escape (38, 41). This may help explain why PD-L1 status alone was not a strong independent discriminator in the present study, whereas a broader phenotype incorporating both local and systemic immune signals was more informative (42).

From a translational perspective, these findings are attractive because both inputs of the integrated phenotype are readily accessible (9). Stromal TILs can be evaluated on routine pathological sections using standardized recommendations from the International Immuno-Oncology Biomarker Working Group, and SII can be derived from routine blood counts without additional cost or specialized platforms. In settings where multigene assays or multiplex immune profiling are unavailable, a simple composite marker based on TILs and SII may therefore offer a pragmatic method to enrich postoperative risk assessment. This is not to suggest that such a phenotype should immediately guide treatment escalation on its own. Rather, our data support its role as an adjunct to standard clinicopathological evaluation and as a candidate variable for future externally validated prognostic tools.

Several limitations should be acknowledged. First, this was a retrospective single-center study, which introduces the possibility of selection bias and center-specific practice effects. Second, although the event count was adequate for exploratory modeling, the cohort size still limits the stability of subgroup analyses, particularly for molecular subtype-specific inference and for smaller pathological strata. Third, although SII was a clinically practical and biologically relevant composite inflammatory index, its optimal categorical threshold has not been standardized in breast cancer, and previously reported cutoffs vary considerably across studies. We therefore used the cohort median for the primary categorical analysis to reduce overfitting and preserve a transparent, reproducible grouping strategy, while also analyzing SII as a continuous variable using restricted cubic splines. Fourth, the assessment of the tumor immune microenvironment remained intentionally focused rather than comprehensive. Stromal TIL evaluation provided a practical measure of local immune infiltration, and the IHC validation used CD8 and CD163 as pragmatic markers of cytotoxic T-cell activity and macrophage-dominant immune suppression. However, this focused panel does not capture the full complexity of the breast cancer immune microenvironment, including total macrophage burden, B-cell compartments, or regulatory T-cell populations. Additional markers such as CD68, CD20, and FOXP3, ideally combined with spatial or multiplex approaches, would provide a more comprehensive translational characterization in future studies. Finally, although the IHC validation supported the biological plausibility of the integrated phenotype, it was performed in representative samples and should be interpreted as focused tissue-level support rather than exhaustive immune profiling.

In conclusion, our study suggests that recurrence prediction in breast cancer can be improved by integrating machine-learning survival modeling with biologically grounded immune-inflammatory markers. RSF achieved the best predictive performance, likely because it captured nonlinear and interacting effects that were incompletely represented by conventional models. At the biological level, SII and the integrated immune phenotype emerged as more informative prognostic signals than stromal TILs alone in this heterogeneous cohort. At the translational level, the favorable and poor phenotypes corresponded to distinct tissue immune states defined by CD8+ and CD163+ infiltration. Together, these findings support a practical framework in which local immune infiltration and systemic inflammatory burden are considered jointly, rather than separately, for DFS risk stratification after breast cancer surgery.

## Data Availability

The original contributions presented in the study are included in the article/**Supplementary Material**. Further inquiries can be directed to the corresponding authors.
