# Supplementary material for: Machine learning-driven identification and immunohistochemical validation of an integrated immune-inflammatory phenotype for disease-free survival stratification in breast cancer
Source: Front Immunol. 2026 Jun 18;17:1836156. doi: 10.3389/fimmu.2026.1836156 (PMC13323226; doi:10.3389/fimmu.2026.1836156)
Supplement: Supplementary file 1 [file Table1.docx]

**Supplementary Table S1. Additional baseline characteristics not shown in Table 1, according to disease-free survival event status.**

| **Characteristic** | **Overall**  N = 503^1^ | **No event**  N = 396^1^ | **Event**  N = 107^1^ | ***p*-value**^2^ |
| --- | --- | --- | --- | --- |
| **Ki-67, %** | 29.00 (17.00, 41.00) | 28.00 (17.00, 40.00) | 31.00 (19.00, 44.00) | 0.12 |
| **Neutrophils, ×10^9/L** | 3.76 (3.13, 4.34) | 3.73 (3.11, 4.23) | 3.85 (3.25, 4.55) | 0.090 |
| **Lymphocytes, ×10^9/L** | 1.96 (1.61, 2.25) | 1.97 (1.65, 2.26) | 1.85 (1.50, 2.21) | 0.10 |
| **Platelets, ×10^9/L** | 254.00 (221.00, 290.00) | 253.00 (222.00, 286.00) | 267.00 (215.00, 301.00) | 0.12 |
| **Albumin, g/L** | 42.70 (40.80, 44.80) | 42.70 (40.90, 44.70) | 42.70 (40.20, 45.10) | 0.7 |
| **C-reactive protein, mg/L** | 2.73 (1.79, 3.86) | 2.70 (1.71, 3.85) | 2.86 (1.86, 4.00) | 0.2 |
| **Neutrophil-to-lymphocyte ratio** | 1.89 (1.54, 2.46) | 1.86 (1.49, 2.43) | 2.05 (1.76, 2.66) | 0.004 |
| **Platelet-to-lymphocyte ratio** | 131.22 (106.64, 165.15) | 127.25 (104.59, 161.58) | 145.22 (112.23, 175.48) | 0.004 |
| **Systemic inflammation response index** | 0.89 (0.66, 1.26) | 0.87 (0.65, 1.21) | 1.02 (0.74, 1.36) | 0.005 |
| **Prognostic nutritional index** | 52.45 (50.10, 54.80) | 52.58 (50.20, 54.95) | 52.10 (49.80, 54.60) | 0.2 |
| **Charlson comorbidity index ≥1** | 115 (23%) | 85 (21%) | 30 (28%) | 0.2 |
| **Histology** |  |  |  | 0.085 |
| Invasive ductal carcinoma | 424 (84%) | 331 (84%) | 93 (87%) |  |
| Invasive lobular carcinoma | 38 (7.6%) | 35 (8.8%) | 3 (2.8%) |  |
| Other invasive carcinoma | 41 (8.2%) | 30 (7.6%) | 11 (10%) |  |
| **ER status** |  |  |  | 0.5 |
| Negative | 105 (21%) | 80 (20%) | 25 (23%) |  |
| Positive | 398 (79%) | 316 (80%) | 82 (77%) |  |
| **PR status** |  |  |  | 0.4 |
| Negative | 223 (44%) | 172 (43%) | 51 (48%) |  |
| Positive | 280 (56%) | 224 (57%) | 56 (52%) |  |
| **HER2 status** |  |  |  | 0.5 |
| Negative | 389 (77%) | 309 (78%) | 80 (75%) |  |
| Positive | 114 (23%) | 87 (22%) | 27 (25%) |  |

^1^Median (Q1, Q3); n (%)

^2^Wilcoxon rank sum test; Pearson's Chi-squared test

**Abbreviations:** DFS, disease-free survival; ER, estrogen receptor; PR, progesterone receptor; HER2, human epidermal growth factor receptor 2; CRP, C-reactive protein; NLR, neutrophil-to-lymphocyte ratio; PLR, platelet-to-lymphocyte ratio; SIRI, systemic inflammation response index; PNI, prognostic nutritional index.

**Supplementary Table S2. Baseline characteristics according to integrated immune phenotype.**

| **Characteristic** | **Overall**  N = 503^1^ | **Favorable**  N = 120^1^ | **Intermediate**  N = 241^1^ | **Poor**  N = 142^1^ | ***p*-value**^2^ |
| --- | --- | --- | --- | --- | --- |
| **Age, years** | 53.49 ± 10.89 | 54.32 ± 10.70 | 53.13 ± 10.92 | 53.38 ± 11.03 | 0.5 |
| **Body mass index, kg/m²** | 24.83 ± 3.62 | 24.93 ± 3.78 | 24.94 ± 3.42 | 24.56 ± 3.82 | 0.7 |
| **Tumor size, cm** | 2.50 (1.90, 3.24) | 2.55 (2.22, 3.38) | 2.45 (1.83, 3.13) | 2.48 (1.82, 3.25) | 0.2 |
| **Stromal TILs, %** | 18.00 (11.00, 27.00) | 29.00 (23.00, 35.00) | 18.00 (11.00, 27.00) | 11.00 (6.00, 16.00) | <0.001 |
| **Systemic immune-inflammation index** | 477.80 (370.42, 633.97) | 372.33 (303.10, 421.63) | 464.44 (338.54, 612.94) | 632.03 (556.24, 793.21) | <0.001 |
| **Menopausal status** |  |  |  |  | 0.4 |
| Premenopausal | 210 (42%) | 44 (37%) | 103 (43%) | 63 (44%) |  |
| Postmenopausal | 293 (58%) | 76 (63%) | 138 (57%) | 79 (56%) |  |
| **Pathological T stage** |  |  |  |  | 0.2 |
| T1 | 144 (29%) | 25 (21%) | 76 (32%) | 43 (30%) |  |
| T2 | 357 (71%) | 94 (78%) | 164 (68%) | 99 (70%) |  |
| T3-4 | 2 (0.4%) | 1 (0.8%) | 1 (0.4%) | 0 (0%) |  |
| **Pathological N stage** |  |  |  |  | 0.4 |
| N0 | 335 (67%) | 80 (67%) | 168 (70%) | 87 (61%) |  |
| N1 | 83 (17%) | 20 (17%) | 35 (15%) | 28 (20%) |  |
| N2 | 57 (11%) | 10 (8.3%) | 27 (11%) | 20 (14%) |  |
| N3 | 28 (5.6%) | 10 (8.3%) | 11 (4.6%) | 7 (4.9%) |  |
| **Histologic grade** |  |  |  |  | 0.002 |
| 1 | 47 (9.3%) | 8 (6.7%) | 24 (10.0%) | 15 (11%) |  |
| 2 | 245 (49%) | 47 (39%) | 113 (47%) | 85 (60%) |  |
| 3 | 211 (42%) | 65 (54%) | 104 (43%) | 42 (30%) |  |
| **Molecular subtype** |  |  |  |  | <0.001 |
| HR+/HER2- | 284 (56%) | 46 (38%) | 135 (56%) | 103 (73%) |  |
| HER2+ | 114 (23%) | 34 (28%) | 48 (20%) | 32 (23%) |  |
| TNBC | 105 (21%) | 40 (33%) | 58 (24%) | 7 (4.9%) |  |
| **Ki-67 category** |  |  |  |  | 0.087 |
| Low | 153 (30%) | 28 (23%) | 74 (31%) | 51 (36%) |  |
| High | 350 (70%) | 92 (77%) | 167 (69%) | 91 (64%) |  |
| **Lymphovascular invasion** | 86 (17%) | 14 (12%) | 39 (16%) | 33 (23%) | 0.040 |
| **PD-L1 status** |  |  |  |  | <0.001 |
| Negative | 404 (80%) | 79 (66%) | 200 (83%) | 125 (88%) |  |
| Positive | 99 (20%) | 41 (34%) | 41 (17%) | 17 (12%) |  |
| **Received neoadjuvant therapy** | 147 (29%) | 43 (36%) | 70 (29%) | 34 (24%) | 0.11 |
| **Breast surgery** |  |  |  |  | 0.2 |
| Breast-conserving | 122 (24%) | 26 (22%) | 67 (28%) | 29 (20%) |  |
| Mastectomy | 381 (76%) | 94 (78%) | 174 (72%) | 113 (80%) |  |
| **Adjuvant chemotherapy** | 462 (92%) | 114 (95%) | 218 (90%) | 130 (92%) | 0.3 |

^1^Median (Q1, Q3) or mean ± standard deviation; n (%)

^2^Kruskal-Wallis rank sum test; Pearson's Chi-squared test

**Abbreviations:** TILs, tumor-infiltrating lymphocytes; SII, systemic immune-inflammation index; PD-L1, programmed death-ligand 1.

**Supplementary Table S3. Subgroup analysis of poor versus favorable integrated immune phenotype for disease-free survival**.

| **Subgroup** | **Level** | **N** | **Events** | **HR (95% CI)** | ***p* value** | ***p* for interaction** |
| --- | --- | --- | --- | --- | --- | --- |
| **Overall** |  | 503 | 107 | 2.53 (1.39–4.60) | 0.002 |  |
| **Pathological N stage** |  |  |  |  |  | **0.397** |
|  | N0 | 335 | 49 | 3.44 (1.43–8.28) | 0.006 |  |
|  | N1 | 83 | 22 | 2.55 (0.59–11.12) | 0.211 |  |
|  | N2 | 57 | 26 | 2.21 (0.43–11.44) | 0.344 |  |
|  | N3 | 28 | 10 | 1.74 (0.06–50.19) | 0.748 |  |
| **Molecular subtype** |  |  |  |  |  | **0.870** |
|  | HR+/HER2- | 284 | 55 | 3.30 (1.12–9.72) | 0.030 |  |
|  | HER2+ | 114 | 27 | 2.33 (0.78–6.91) | 0.128 |  |
|  | TNBC | 105 | 25 | 3.74 (0.66–21.12) | 0.135 |  |
| **Breast surgery** |  |  |  |  |  | **0.334** |
|  | Breast-conserving | 122 | 22 | 15.73 (2.66–93.04) | 0.002 |  |
|  | Mastectomy | 381 | 85 | 2.02 (1.06–3.85) | 0.032 |  |
| **Adjuvant chemotherapy** |  |  |  |  |  | **0.565** |
|  | No | 41 | 6 | — | — |  |
|  | Yes | 462 | 101 | 2.52 (1.36–4.67) | 0.003 |  |
| **Neoadjuvant therapy** |  |  |  |  |  | **0.881** |
|  | No | 356 | 79 | 2.73 (1.32–5.67) | 0.007 |  |
|  | Yes | 147 | 28 | 1.91 (0.63–5.77) | 0.253 |  |

**Notes:** Hazard ratios compare the poor integrated immune phenotype with the favorable phenotype. Subgroup-specific hazard ratios were estimated using Cox proportional hazards models adjusted according to the subgroup-analysis framework, excluding the stratification variable itself within each subgroup. *p* for interaction values were derived from likelihood ratio tests comparing models with and without the interaction term. Results should be interpreted as exploratory.

**Abbreviations:** HR, hazard ratio; CI, confidence interval; TNBC, triple-negative breast cancer.
